# Pop-In Phenomenon as a Fundamental Plasticity Probed by Nanoindentation Technique

**DOI:** 10.3390/ma14081879

**Published:** 2021-04-09

**Authors:** Takahito Ohmura, Masato Wakeda

**Affiliations:** Research Center for Structural Materials, National Institute for Materials Science, 1-2-1 Sengen, Tsukuba 305-0047, Japan; WAKEDA.Masato@nims.go.jp

**Keywords:** pop-in, nanoindentation, plasticity initiation, dislocation nucleation, lattice defects

## Abstract

The attractive strain burst phenomenon, so-called “pop-in”, during indentation-induced deformation at a very small scale is discussed as a fundamental deformation behavior in various materials. The nanoindentation technique can probe a mechanical response to a very low applied load, and the behavior can be mechanically and physically analyzed. The pop-in phenomenon can be understood as incipient plasticity under an indentation load, and dislocation nucleation at a small volume is a major mechanism for the event. Experimental and computational studies of the pop-in phenomenon are reviewed in terms of pioneering discovery, experimental clarification, physical modeling in the thermally activated process, crystal plasticity, effects of pre-existing lattice defects including dislocations, in-solution alloying elements, and grain boundaries, as well as atomistic modeling in computational simulation. The related non-dislocation behaviors are also discussed in a shear transformation zone in bulk metallic glass materials and phase transformation in semiconductors and metals. A future perspective from both engineering and scientific views is finally provided for further interpretation of the mechanical behaviors of materials.

## 1. Introduction

Mechanical property testing by indentation-induced deformation has a long history predating 1900. Hardness testing is one of the most useful and reliable methods to evaluate the macroscopic strength of materials because of its simple protocols, including easy sample preparation and high-throughput testing. Therefore, hardness testing has been used as a substitute for tensile/compression testing and as a non-destructive evaluation method. To obtain a higher quantitative evaluation in engineering applications, Tabor established the equation, *H* = 3*σ*_f_, in 1951 [1], where *H* is the indentation hardness and *σ*_f_ is the flow stress. Since then, indentation techniques have been used on various materials, not only metallic ones, but also on relatively brittle materials including ceramics, semiconductors, and intermetallic compounds.

Another advantage of the indentation technique is the minimization of the tested area of a material to probe each microstructural component and separate an individual contribution to the mechanical properties for further interpretation of the strengthening factors and mechanisms. Structural materials in all constructions are used on a millimeter or larger scale, while the microstructures in materials are designed and controlled on the nanometer to micrometer scale. Therefore, mechanical characterization at the same microstructural scale is critical to improve the guiding principles of material design to obtain better-performing materials. A greater demand for improved performance from materials has driven the dimensional reduction of the microstructures, including film thickness and particle size; hence, mechanical characterization techniques have improved measurement accuracy at smaller scales to meet this demand.

Nanoindentation is one of the most rapidly developing techniques for the characterization of fine microstructures. The nanoindentation method pushes an indenter into a sample surface under a μN resolution load and measures the penetration depth, in nanometers, to evaluate the elastoplastic deformation of materials. The indentation depth and horizontal length are typically less than 100 nm and less than a micron, respectively. The depth is measured using a displacement gauge and then converted into the contact area by using the geometry of the indenter. It can be called depth-sensing indentation, based on the measurement principle. Details of the principles of this technique are available in the literature [2,3,4,5,6,7,8,9,10,11,12,13]. The nanoindentation technique is just on the milestone of the 30-year anniversary of the publication of the landmark paper by Oliver and Pharr in 1992 [7]. Instrumented indentation techniques are already established as one of the most useful and powerful tools for the mechanical testing of materials. As material design technology advances into ever smaller scales, finer microstructures, and multielement structures, a strong demand exists to further evolve these techniques to achieve higher performance, even in harsh environments; for example, at elevated temperatures.

In addition to industrial engineering applications, indentation techniques have been used in fundamental science, including atomic-scale modeling of the elemental steps of deformation and fracture. Continuum mechanics is a major approach in the conventional modeling of the deformation of materials, especially for the macroscopic behavior of materials, and one of the useful theories is mean field approximation. In contrast, the mechanical behavior can be probed with much higher resolution, both on a spatial scale and in a time period, by improving the instrumentation in the indentation techniques. The advanced technique enables us to obtain not only the statistical, but also dynamic behavior of materials, and has other approaches including crystal plasticity with dislocation theory and quantum modeling. The strain burst phenomenon under an indentation-loaded condition, so-called “*pop-in*”, is an attractive phenomenon for every science field, including physics, mechanics, and materials science. Pop-in is understood as an incipient plasticity, as described in the subsequent sections; therefore, it is very important to reveal the mechanisms of the yielding and factors of the yield strength of materials, which is absolutely necessary for designing practical structural components for social infrastructures and transport equipment. Furthermore, incipient plasticity involves the activation and/or generation of dislocations, and subsequent burst events are associated with the unstable phenomenon, which is one of the most interesting issues in solid-state physics.

This paper reviews the experimental and computational approaches to the pop-in phenomenon and discusses the fundamental mechanical behavior mechanisms of various materials for a deeper understanding of the strengthening factors of the macroscopic properties.

## 2. Pioneering Works

A characteristic phenomenon during nanoindentation, especially in a loading segment, is incipient plasticity at extremely high stress levels close to the theoretical strength. Gane and Bowden first found the phenomenon for the face-centered cubic (fcc) metals of Au, Cu, and Al [14]. They conducted a novel experimental technique of in situ point loading with a sharp stylus in a scanning electron microscope to reveal the critical stress for plasticity initiation at a theoretical strength. The contact size was on the order of 100 nm, which could be considerably smaller than the average spacing of dislocations; hence, the onset of plasticity occurred in a perfect crystal. Even though their apparatus could not measure any load–displacement curve at that time, they suggested a strain-burst like behavior with the description: “*Instead of the indentation size increasing as the load was increased, no indentation was observed until a critical load was reached. The stylus then suddenly penetrated the surface to produce an indentation*”. The sudden penetration could be a strain burst phenomenon, subsequently termed “pop-in” behavior. Interestingly, they said, “*The reason for this is not yet clear*,” while also suggesting, “*We may, in fact, be measuring the stress necessary to create dislocations in a perfect crystal lattice*”. It is surprising that the phenomenon was predicted more than 50 years ago. The subsequent works of Gane demonstrated further that the theoretical stress can be also detected in non-metallic materials [15], and the phenomenon occurs only at very low load levels [16]. Another group subsequently demonstrated “yielding” in body-centered cubic (bcc) metals [7], and molecular dynamics (MD) simulations were used to interpret the behavior on an atomistic scale [17,18]. Two decades after the pioneering work by Gane and Bowden in 1968, MacMillan demonstrated plasticity initiation at a theoretical stress with an analysis of a load, *P,* against displacement, *h,* curve [19]. He fitted a Hertz contact curve to a measured *P*–*h* curve to determine the onset of plasticity at the *P*–*h* data deviation from the Hertz curve.

**Figure 1:**
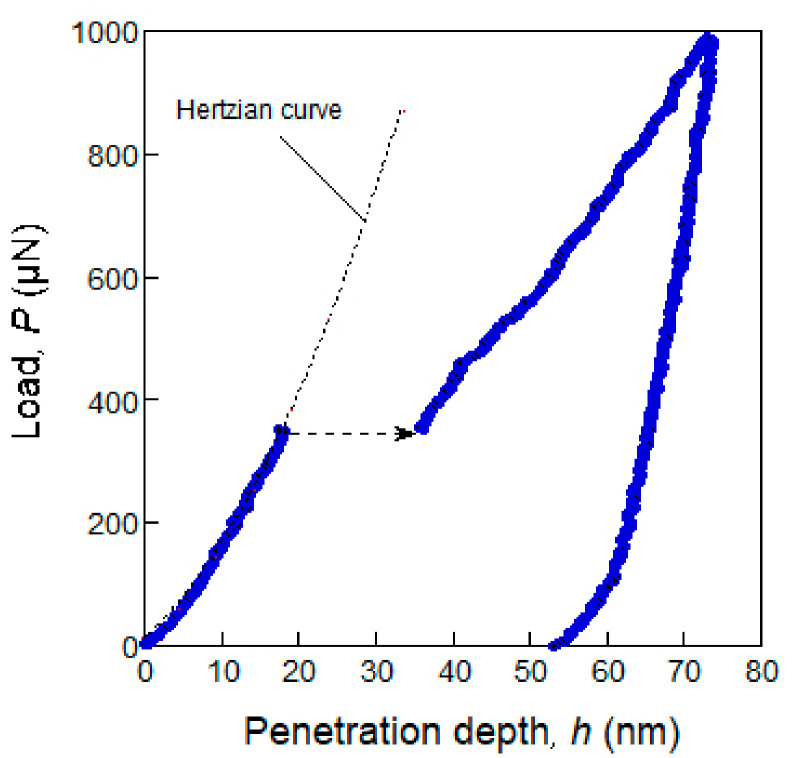
Typical load–displacement curve for an Fe alloy showing the pop-in phenomenon on the loading curve, indicated by the dashed-line arrow. The broken line represents a Hertzian curve fitted with the experimental data.

The principle of the theoretical stress analysis is described quantitatively in an Fe alloy, with Figure 1 as an example. When *P_c_* is defined as a critical load for a pop-in event, the load–displacement curve that is lower than *P_c_* fits very well with the dashed line of the Hertz contact model [20], given as:

(1) $$P=\frac{4}{3}E_{r}R^{\frac{1}{2}}h^{\frac{3}{2}}$$

where *R* is the indenter tip radius of curvature and *E_r_* is the reduced modulus, given as:

(2) $$\frac{1}{E_{r}}=\frac{1-\nu_{i}{}^{2}}{E_{i}}+\frac{1-\nu_{s}{}^{2}}{E_{s}}$$

where *E* and *ν* are the Young’s modulus and Poisson’s ratio, respectively, and the subscripts *i* and *s* refer to the indenter and sample, respectively. This result clearly indicates that deformation before the pop-in event is dominated by purely elastic deformation. Additionally, the maximum shear stress beneath the indenter, *τ*_max_, is expressed as follows:

(3) $$\tau_{max}=0.18{\left(\frac{E_{r}}{R}\right)}^{\frac{2}{3}}P^{\frac{1}{3}}$$

when *P_c_* = 350 μN, as shown in Figure 1, and *P* was substituted into Equation (3); *τ*_max_ was calculated as 11.3 GPa, which is approximately 1/7th of the 83 GPa shear modulus of the ideal strength.

## 3. Experimental Clarification

To experimentally locate the theoretical strength under a very low load at the point contact condition, Pethica and Tabor first claimed that the ideal strength can be obtained under, and subject to, the existence of an approximately 5 nm-thick oxide layer on the sample surface of Ni [21]. Venkataraman et al. and Gerberich et al. subsequently demonstrated in an Fe–3Si alloy that the theoretical strength could be obtained with a passive oxide layer on the sample surface, while the strength reduced by two orders after removing the oxide layer [22,23]. Chechenin et al. also demonstrated the effect of a surface oxide layer and suggested that the initiation corresponds to a break in the oxide layer [24]. They used the word “pop-in” to mean incipient plasticity, possibly for the first time in the literature. Gerberich et al. presented a model of the effect of the oxide layer with a two-step mechanism; that is, dislocation nucleation below the oxide layer for the first step and rupture of the oxide layer by the nucleated dislocation gride for the second step [25,26,27,28]. Mann and Pethica showed the loading rate dependency of the pop-in behavior for GaAs and W, indicating that the dependence is strongly affected by surface chemistry [29]. In contrast, Corcoran et al. demonstrated another experimental result for Au single crystals, claiming that the pop-in phenomenon can occur at a theoretical stress level even in the absence of a native oxide layer on the surface [30]. Asif and Pethica showed that no plastic deformation occurs below the pop-in load for an electropolished W, suggesting a creatin of mobile dislocation upon pop-in [31]. Barnoush and Corcoran subsequently demonstrated corresponding results for Al alloys [32]. The results included the effects of the pre-existing dislocation density and curvature of an indenter. Homogeneous dislocation nucleation occurs when the indentation stress is mainly induced within a defect-free region under conditions of relatively low dislocation density, indicating that the rupture of native oxide is not necessarily a unique mechanism.

**Figure 2:**
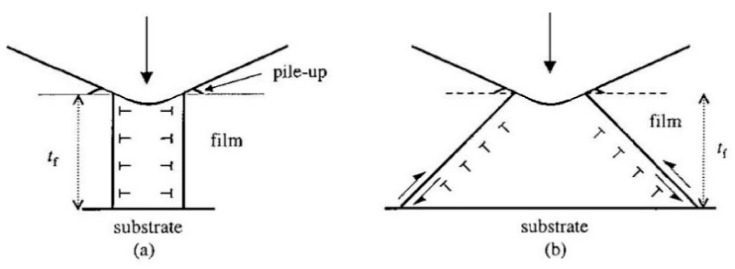
Two scenarios for dislocation nucleation beneath an indenter. (**a**) Punching of a PDL with a diameter equal to the indenter sample contact area, and (**b**) shear-band formation with a shear dislocation loop (including a half one) nucleating and gliding into a deeper region [33].

Other experimental approaches were used to understand the elementary steps of incipient plasticity based on the dislocation theory. Suresh et al. demonstrated that the critical stress of the onset of plasticity is close to the theoretical strength and does not depend on the film thickness in Cu thin films, indicating plasticity initiation with dislocation nucleation at the defect-free volume [33]. They proposed two scenarios of dislocation nucleation beneath the indenter, as shown in Figure 2. One is the punching of a prismatic dislocation loop (PDL) with a diameter equal to the indenter sample contact area. The other is a shear-band formation with a shear dislocation loop (including a half one) nucleating and gliding into a deeper region. Gouldstone et al. discussed an energetic model for dislocation emission beneath an indenter [34]. They estimated the stored elastic strain energy in a sample immediately before the strain burst by the integration of a parabolic *P*–*h* curve, and the summation of the self-energy of the PDL and the elastic interaction between them after the event. When the estimated energy values were balanced by a fitting parameter of the diameter of the PDL, the computed diameter was roughly coincident with the horizontal size of the contact area between the indenter and sample, suggesting that the PDL generation model is a reasonable mechanism.

**Figure 3:**
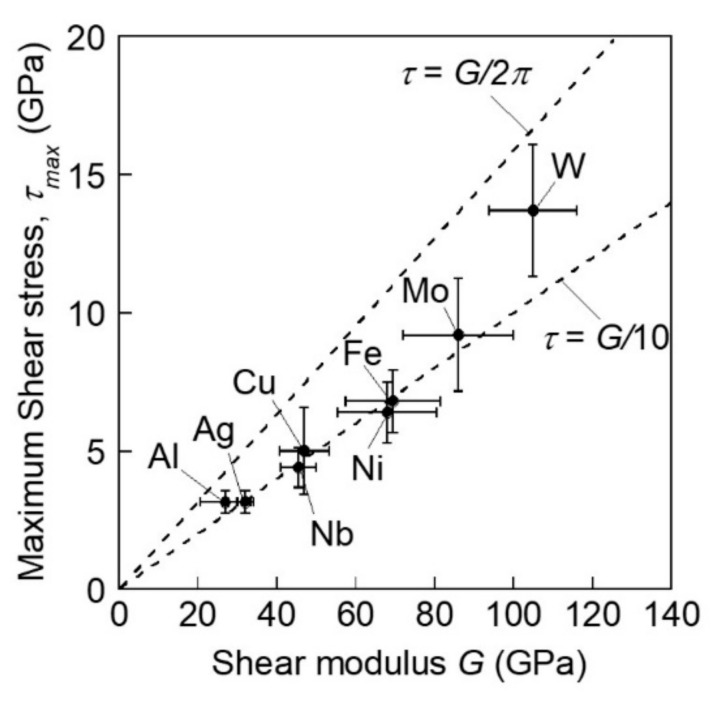
Relationship between the maximum shear stress, *τ*_max_, calculated from the pop-in load, *P*_c_, using Equation (3) and the stiffness modulus, *G,* converted from the Young’s modulus calculated from the unloading curve [36].

Gerberich et al. also proposed an energetic consideration for incipient plasticity [35]. They discussed two cases; that is, single-loop nucleation in the absence of oxide film fracture and multiple dislocation avalanches in the presence of an oxide film fracture. In the former case of single-loop nucleation, the work by the applied load can be accounted by dislocation and surface work, as well as stored elastic strain energy. The latter case should be modeled based on an instability phenomenon with a balance in the energy between the plastic deformation energy and surface energy. These theoretically based models provide a practical consideration of material behavior. Ohmura et al. conducted a systematic analysis of various single crystals from a variety of materials with different crystal structures [36]. All vertical directions of the sample surface were oriented to <001>. Figure 3 shows the relationship between the maximum shear stress, *τ*_max_, calculated from the pop-in load, *P*_c_, using the Hertz model in Equation (3), and the stiffness modulus, *G*, converted from the Young’s modulus calculated from the unloading curve. This relation was linear for all the measured materials, and the coefficient was found to be approximately 1/2π. In contrast, one of the models in which the frictional stress of the perfect crystal on the slip plane is formulated in a simple Peierls stress is given as [37]:

(4) $$\tau =\frac{b}{d}\frac{G}{2\pi }\sin\left(\frac{2\pi x}{b}\right)$$

where *b* is the magnitude of the Burgers vector, *d* is the distance between the slip planes, and *x* is the relative displacement in the slip direction. The maximum stress obtained by approximating *b* to *d* is G/2π when *x* = *b*/4. This value approximates that obtained experimentally, as shown in Figure 3. This result strongly indicates that the stress level at which the pop-in behavior appears is close to the ideal strength regardless of the crystal structure, indicating that the critical stress strongly depends on the local shear modulus. Remington et al. proposed another model based on a combination of experimental and computational simulations [38]. They showed a unique mechanism of forming a PDL by an interaction between shear half loops that are generated in fast plasticity.

**Figure 4:**
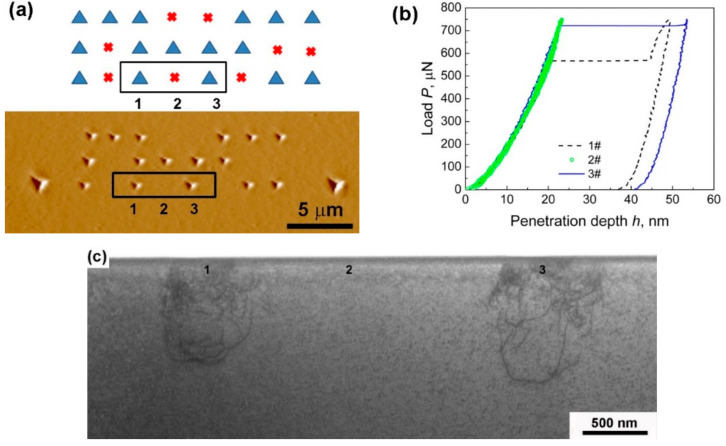
(**a**) SPM image of indentation marks on the sample surface, (**b**) the corresponding load–displacement curves, and (**c**) the cross-sectional TEM images of the dislocation structures just below the indentation marks [39].

Zhang et al. demonstrated dislocation structures beneath the indenter using a transmission electron microscope (TEM) image [39]. They conducted indentation testing on an Fe–Si sample in an array with the same peak load conditions, and then a TEM sample in the cross-sectional view beneath the indent marks was selected to observe the dislocation structures after the pop-in event. Figure 4a shows a scanning probe microscope (SPM) image of the sample surface with triangle indent marks and a schematic showing the 3 × 8 array of the indentation positions. Among the 24 positions, some included clear triangle indent marks, while others do not. Three typical positions within a rectangle are shown in the image. The triangle indent marks are seen in positions #1 and #3, whereas no mark is visible at #2. The corresponding *P*–*h* curves for the three cases are shown in Figure 4b. The curves for positions #1 and #3 show clear pop-in events on the loading curve, whereas the unloading curve perfectly overlaps the loading curve for #2, indicating an absolutely elastic deformation. The corresponding TEM image of the dislocation structure beneath the indenter is shown in Figure 4c. High-density dislocation structures were observed at positions #1 and #3, but no dislocations were observed at position #2. It should be noted that in the case of position #3, the unloading started immediately after the pop-in, indicating that the dislocation structure was formed within a very short time of the pop-in event.

**Figure 5:**
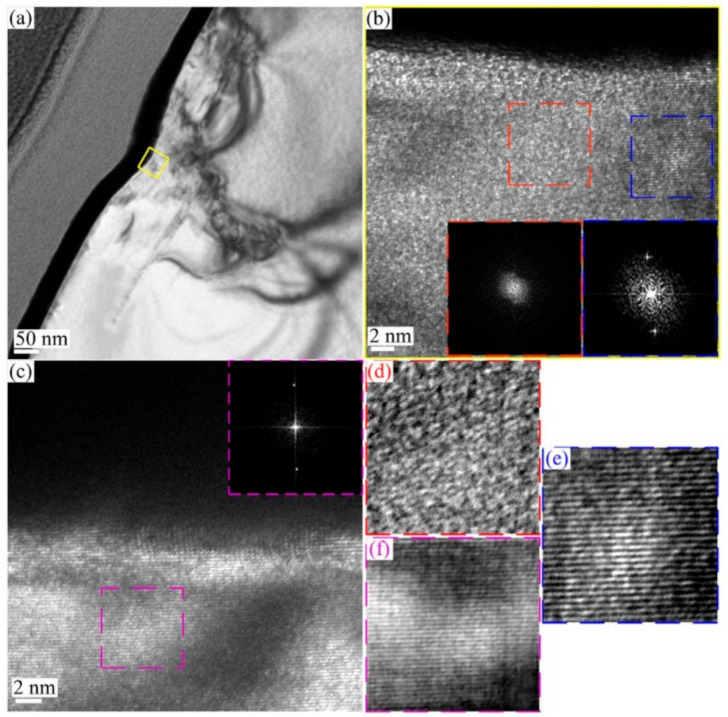
TEM image demonstrating amorphization underneath the indenter in SiC, (**a**) a bright field low-magnification TEM image of the plastically deformed 6H-SiC, (**b**) a high-magnification TEM image taken from the yellow border region of (**a**). (**c**) a high-magnification TEM image taken from region far away from the residual nanoindentation site. A reduced fast Fourier transformation (FFT) was conducted in the three selected regions marked by red, green and purple frames, as shown in the inserts; while the Wiener-filtered images of these regions are shown in (**d**), (**e**) and (**f**), respectively [40].

Wu et al. recently presented an interesting TEM image demonstrating amorphization beneath the indenter in SiC, as shown in Figure 5 [40]. TEM images include high-resolution images of the lattice, indicating an amorphous phase, as shown in Figure 5b. Amorphization beneath the indenter is frequently discussed in semiconductors, including Si [41], in terms of indentation-induced phase transformation, but it is interesting that a material with a stable lattice structure also shows amorphization, suggesting a very complicated mechanism of plasticity initiation. They proposed a scenario of the pop-in event in the two-step event; that is, amorphization for the first pop-in and subsequent dislocation generation and propagation corresponding to the second, or later, events.

**Figure 6:**
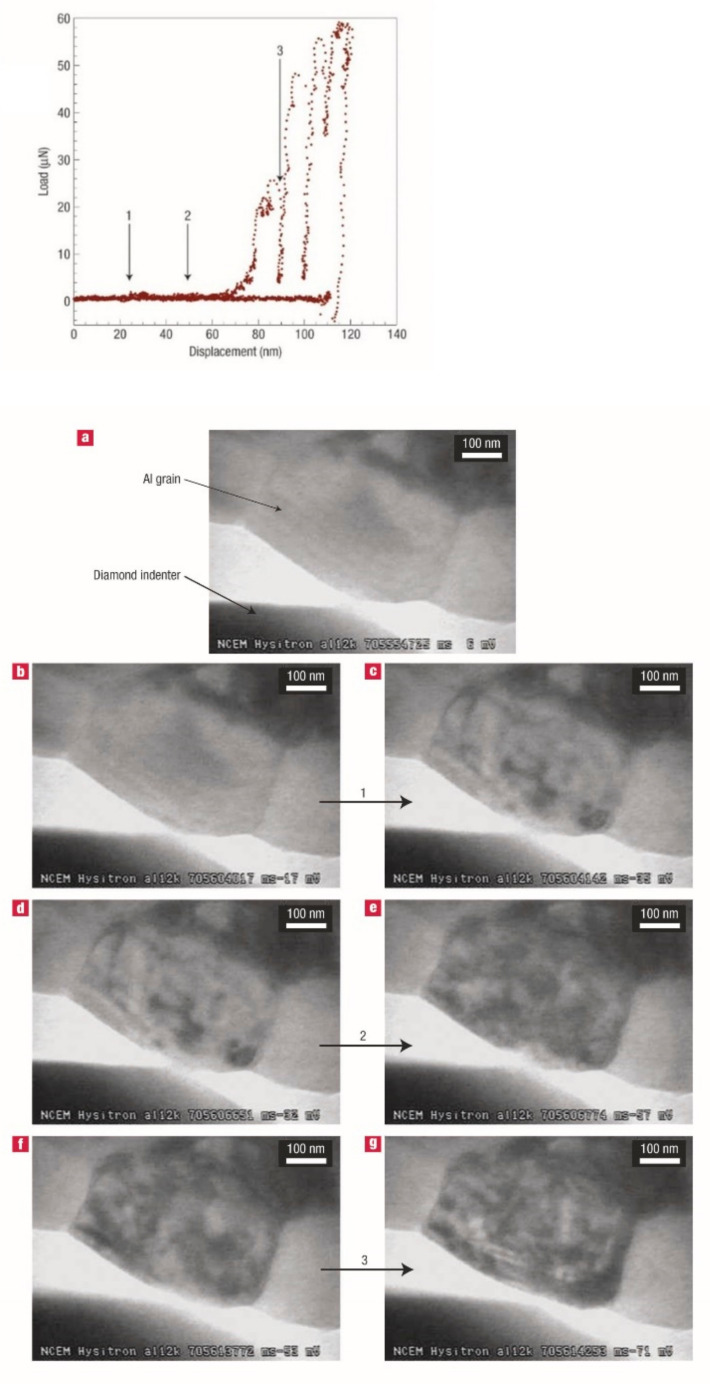
Load–displacement curve (above) and TEM micrographs (bottom) representing the microstructural evolution sequence and the associated mechanical response during TEM in situ straining of Al [42]. The indented grain is initially free of dislocations (**a**), (**b**,**c**), (**d**,**e**), and (**f**,**g**), extracted video frames corresponding to transients arrowed as 1, 2, and 3 in the above load-displacement curve.

Important experimental results have been reported in the literature for the process of forming dislocation structures associated with pop-in events. Minor et al. demonstrated that dislocations were activated prior to a pop-in event on a loading process using a novel technique of TEM in situ indentation for Al [42]. Figure 6 shows the load–displacement curve and TEM micrographs representing the microstructural evolution sequence and the associated mechanical response. The TEM images clearly show dislocation activation, corresponding to positions 1 to 3 on the curve. The dislocation events at 1 and 2 occurred prior to the pop-in event at 3, indicating that dislocations were activated in advance of the strain burst. This is an important work to reveal the pop-in mechanism, and several subsequent studies have shown a similar behavior in various materials [43,44,45,46].

One of the most essential issues for plasticity initiation is the dislocation nucleation mechanism of homogeneous/heterogeneous models. A presumed elementary step for this phenomenon is a thermally activated process. Lorenz et al. suggested that a homogeneous nucleation of a shear loop of a dislocation occurs in a defect-free region beneath the indenter for various materials, including metals, semiconductors, and ionic crystals [47]. Mao et al. also experimentally suggested the homogeneous nucleation of dislocations in polycrystalline and single-crystal alumina [48]. In contrast, Schuh et al. suggested heterogeneous nucleation based on a stress-assisted activation model [49]. They evaluated the activation energy, activation volume, and attempt frequency for Pt samples experimentally at various temperatures and strain rates, and found that these parameters were “*strikingly small*”. They said, “*It is quite unlikely that the present data corresponds to the homogeneous nucleation of a dislocation loop beneath the indenter*”, and suggested a potential vacancy and/or vacancy cluster mechanism. Several subsequent studies have also described the heterogeneous nucleation of dislocations for the initiation of plasticity. Bei et al. suggested that a full dislocation loop homogeneously nucleated in the bulk as opposed to a half or quarter loop being heterogeneously nucleated at the surface or edge of a sample [50]. Wu et al. reported that interstitial atoms cause heterogeneity in bcc chromium [51]. Xia et al. showed the effect of the substitutional alloying element of the Fe–Cr–Ni system on incipient plasticity with a heterogeneous nucleation model [52]. The nucleation of dislocations could be the most fundamental process in the onset of plasticity, and further investigation is important.

Another interesting viewpoint can be given by comparing bcc and fcc structures because the dislocation structure and mobility are considerably different in the two crystallographic structures. Vadalakond et al. compared the strain burst behavior of W, Fe, and Ni, and showed that the frequency of the burst in the early stage of the loading curve, below approximately 0.1 mN, was higher for bcc W and Fe than for fcc Ni [53]. They concluded that bcc metals have a higher number of slip systems (mainly a variety of slip planes) than fcc metals. Biener et al. performed a systematic analysis of bcc Ta and claimed that the excursion depth in the pop-in event was larger in bcc than in fcc [54]. They discussed the reason for the difference in the higher Peierls stress in bcc metals. Additionally, they demonstrated that the critical stress for the onset of plasticity depends on the larger loading rate in bcc than that in fcc, indicating the higher dominance of the applied stress on the stress-biased thermally activated dislocation nucleation process due to the lower mobility of screw dislocations in bcc structures. Another type of nanoscale mechanical characterization in micropillars also provides a deep understanding of the difference in behavior between the two crystals. Brinckmann et al. and Greer et al. discussed the experimental and simulated mechanical behavior of bcc and fcc metals [55,56]. They indicated that dislocation generation occurs preferentially at the sample surface in the case of fcc, whereas source formation by cross slip is dominant in the case of bcc.

The other crystal-plasticity-based issue is dependent on the crystallographic orientation [57,58,59,60,61,62,63,64,65,66,67,68,69,70,71]. Kiely and Houston performed an indentation using an interfacial force microscope on Au single crystals with three orientations and showed that the critical resolved shear stress for incipient plasticity deformation on the {111}<110> slip system was identical in the different indented orientations, even though the critical load varied significantly depending on the indented orientation [57]. Kwon et al. and Catoor et al. analyzed the deformation behavior in hexagonal close-packed α-Ti and Mg, and showed that the preferentially activated slip and twin in the various slip planes of basal, prismatic, and pyramidal can be explained in terms of the indentation Schmid factor [65,66]. These abovementioned arguments on the crystal-plasticity-based dislocation theory are important for interpreting the fundamental deformation behavior in crystalline materials.

Another approach using a spherical indenter tip is a validated methodology for the mechanisms of incipient plasticity [72,73,74,75,76,77]. Michalske and Houston performed a contact experiment using interfacial force microscopy with various tip radii. They demonstrated that the shear stress at the contact point reached a theoretical limit [72]. They showed that the critical stress increased with a decreasing probe-tip radius, which was presumably due to a sample surface effect. Morris et al. also demonstrated the same size effect as the critical stress and proposed a stochastic model of the dependency [76].

The other twin and crack formation mechanisms are discussed in the transition from pure elastic to other behaviors. Twin formation upon plasticity initiation has been reported in sapphire [78], high-Mn steel [79], Ta [80], Mg–6Zn [81], and nanocrystalline (nc)-Fe [82]. Crack initiation can also be the dominant mechanism for the event, as shown mainly in brittle materials, including polycrystalline alumina [83], TiN-based thin-film coatings [84,85], Si and Ge [86,87], tungsten carbide [88], and various ceramics [89]. Twining and cracking always compete with dislocation glide in various materials, and it is important to consider every mechanism for the interpretation of the material behavior.

Nanoindentation and combined experimental approaches are a powerful methodology to probe the fundamental and local mechanical behaviors, as reviewed in this section. For further interpretation of the deformation mechanisms, we should improve the techniques to vary measurement circumstances including temperatures, atmospheres, solutions, and so on. A cryogenic condition is one of the most imperative approaches because the thermally activated process could be a dominant mechanism, as described in the subsequent section, and hence becomes conspicuous at lower temperatures. The atmospheric control is also essential for not only the basic point of view, but also industrial applications such as hydrogen embrittlement.

## 4. Physical Modeling

**Figure 7:**
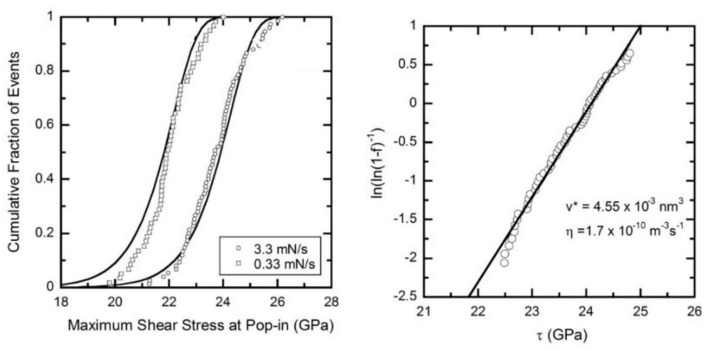
A cumulative proverbiality function and the estimated activation volume in TiC [94].

The nanoindentation-induced pop-in phenomenon has motivated the understanding of the fundamental aspects of plasticity initiation at the nanoscale with defect-free volumes. Because the stress for the event is close to the theoretical strength, as suggested by the papers mentioned above, it is presumed that an elementary step of the plasticity initiation is associated with the nucleation of dislocations. Bahr et al. experimentally demonstrated that the incubation time for the strain burst while holding the load in an indentation is longer for a lower applied load, suggesting a stochastic mechanism that is assisted by an applied stress [90]. They proposed a model for the nucleation of a shear dislocation loop for a stochastic event. Kucheyev et al. showed that the pop-in phenomenon was associated with the onset of slip deformation in MgO, and the critical load, *P_c_,* for the event on a loading curve was higher for a higher loading rate [91]. Schuh et al. revealed that in a bulk metallic glass material, the frequency of pop-in occurrence was higher under a lower strain rate [92]. Their results indicate a kinetic effect on the phenomenon and correspond to the conclusion given by Bahr. Chiu et al. also studied incipient plasticity in a Ni_3_Al single crystal [93]. They conducted constant-load testing to measure the time for pop-in to occur, and showed that the holding time was longer for the lower applied load. This is the kinetic effect suggested by previous studies, but the authors of this paper discussed the diffusion of vacancies as the kinetic process for the “*time-delay effect*” of the load dependency in the pop-in event. After several important experimental works on various materials, Schuh and Lund proposed a stress-biased, thermally activated model based on the nucleation theory by using a cumulative proverbiality function and estimated the activation volume in TiC, as shown in Figure 7 [94]. This model provides a chance to consider the detailed mechanism of the critical event. Schuh and Lund also demonstrated that the estimated activation volume was less than 1.0 *b*^3^, where *b* is the magnitude of the Burgers vector, which is considerably smaller than the values measured in conventional methods, including tensile testing, suggesting heterogeneous nucleation rather than homogeneous nucleation beneath the indenter.

Subsequently, several studies discussed the physical model of incipient plasticity [95,96,97,98,99,100,101,102,103,104,105,106,107,108,109,110,111]. Mason et al. estimated the activation energy and volume for Pt at various temperatures and strain rates [98]. They estimated the activation energy, activation volume, and attempt frequency for incipient plasticity, and suggested a plausible mechanism for the incipient plasticity of heterogeneous nucleation of dislocations at pre-existing point defects. Paul et al. performed scanning tunneling microscopy (STM) and atomic force microscopy (AFM) experiments for Au with a W spherical indenter, and found that a minimum energy of approximately 70 eV was necessary to generate a minimum plastic deformation after elastic loading [107]. The threshold energy is considerably higher than that of the dislocation glide motion in fcc metals; therefore, the incipient plasticity under a small contact volume has an extremely high energy barrier. Several studies discussed the probability distribution function for the event. Wo et al. measured the time dependency of incipient plasticity in Ni_3_Al and proposed a Poisson-like or exponential distribution function for the phenomenon [97]. Li et al. performed a theoretical study on physical modeling and concluded that the probability function of a thermally activated process was dominant in the case of homogeneous nucleation, but it depended on the spatial stochastic function of pre-existing lattice defects on a larger scale, suggesting the activation of pre-existing dislocations [105]. Somekawa et al. discussed the activation process of Mg [103]. They demonstrated that the strain-rate sensitivity was larger and the activation volume was smaller in the pop-in event than in macroscopic deformation. Based on the experimental results, they concluded that cross slip dominated the deformation at the macroscale, whereas dislocation nucleation occurred in the pop-in phenomenon. Additionally, the alloying effect was small in the pop-in event, but was macroscopically large through a stacking-fault energy. An advanced methodology of indentation testing at elevated temperatures provided clear evidence for the thermally activated process [98,101,102,103,109]. Packard et al. showed a difference in the probability function; that is, it depended on the temperature of the crystalline materials and on the atomic-arrangement structure in amorphous materials [101]. Rajulapati et al. showed a temperature dependence of the pop-in behavior in Ta; that is, a single event with a large magnitude occurred at ambient temperature, while multiple events with smaller magnitudes appeared at elevated temperatures owing to the higher mobility in the dislocation glide motion [102]. Franke et al. also performed a high-temperature experiment, as well as finite element method (FEM) analysis, in Ta single crystals with various crystallographic orientations, and showed that a serrated flow in the *P–h* curve was associated with a specific defect network by quasi-elastic reloading [109]. The thermally or stress-assisted activation of dislocation nucleation has been discussed in various other materials, including intermetallic compounds [99], GaN [106], and multi-principal element alloys [110], indicating that incipient plasticity is governed by universal mechanisms.

The detail in mechanism of dislocation nucleation at defect-free volume is not necessarily covered by the conventional dislocation theory. One of the keys could be an effect of the heterogeneity because every material includes lattice defects to minimize the free energy. This kind of “romantic” topic attracts people in various fields, and many more collaborations between them are expected for a significant progress.

## 5. Effect of Pre-Existing Lattice Defects

### 5.1. Initial Dislocation Density

**Figure 8:**
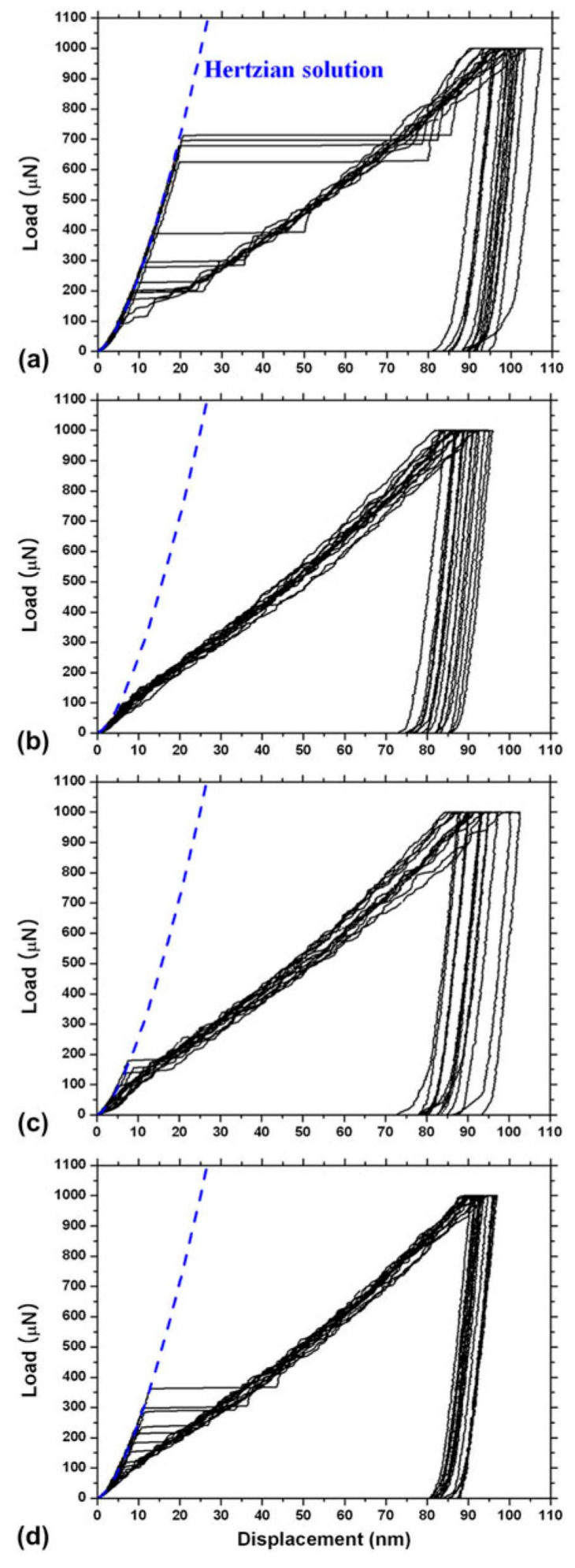
Load–displacement curves for a Fe–C alloy. (**a**) Fully annealed sample; (**b**) after a 6% uniaxial tensile prestrain; (**c**) 30 h after the prestrain; and (**d**) 3 weeks after the prestrain [119].

Pre-existing lattice defects, including dislocations and dislocation sources, affect the behavior of plasticity initiation beneath an indenter [32,112,113,114,115,116,117,118,119,120,121,122,123,124]. Bahr et al. demonstrated that the critical stress for yielding can reach the theoretical strength of W and Fe only under a low density of dislocation source [112]. Miller et al. showed that the pop-in behavior in Au occurs only after annealing, suggesting that a low density of pre-existing dislocations is required for this phenomenon [113]. Ahn et al. demonstrated the effects of dislocation density and strain aging on the pop-in behavior in bcc Fe-C alloys [119]. Figure 8 shows the load–displacement curves of the alloy. Clear pop-in behaviors are visible for the fully annealed sample in Figure 8a. After a 6% uniaxial tensile prestrain, the pop-in phenomenon disappeared immediately, as shown in Figure 8b, indicating that tensile-strain induced dislocations enhanced incipient plasticity. After 30 h, the pop-in occurred again at a considerably lower load, as shown in Figure 8c, and the pop-in critical load and frequency increased further after three weeks, as shown in Figure 8d. The reappearance of the pop-in can be understood as strain aging by solute C atoms segregating at dislocations to form the so-called Cottrell atmosphere.

**Figure 9:**
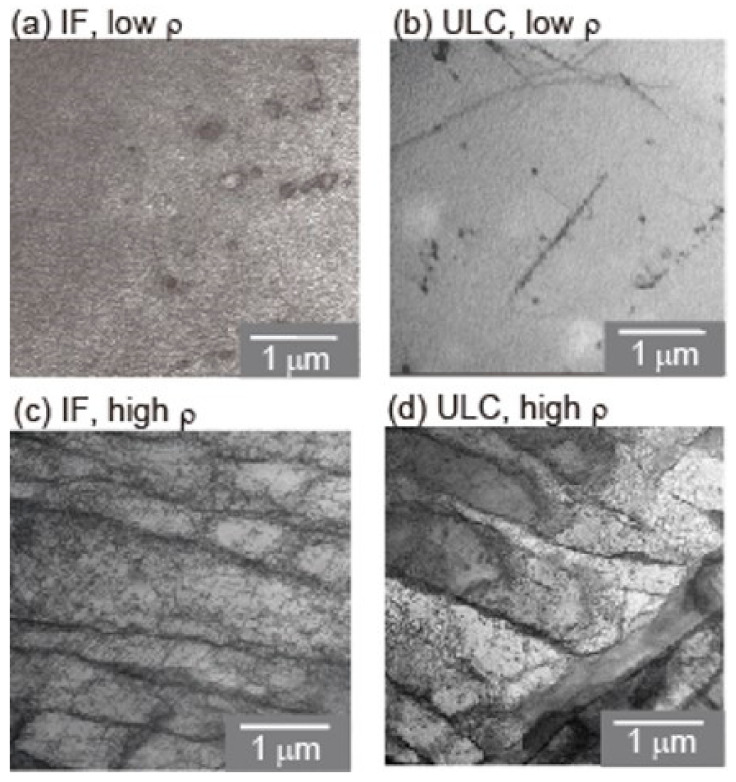
STEM micrographs for interstitial free (IF) steels with different dislocation densities [120].

**Figure 10:**
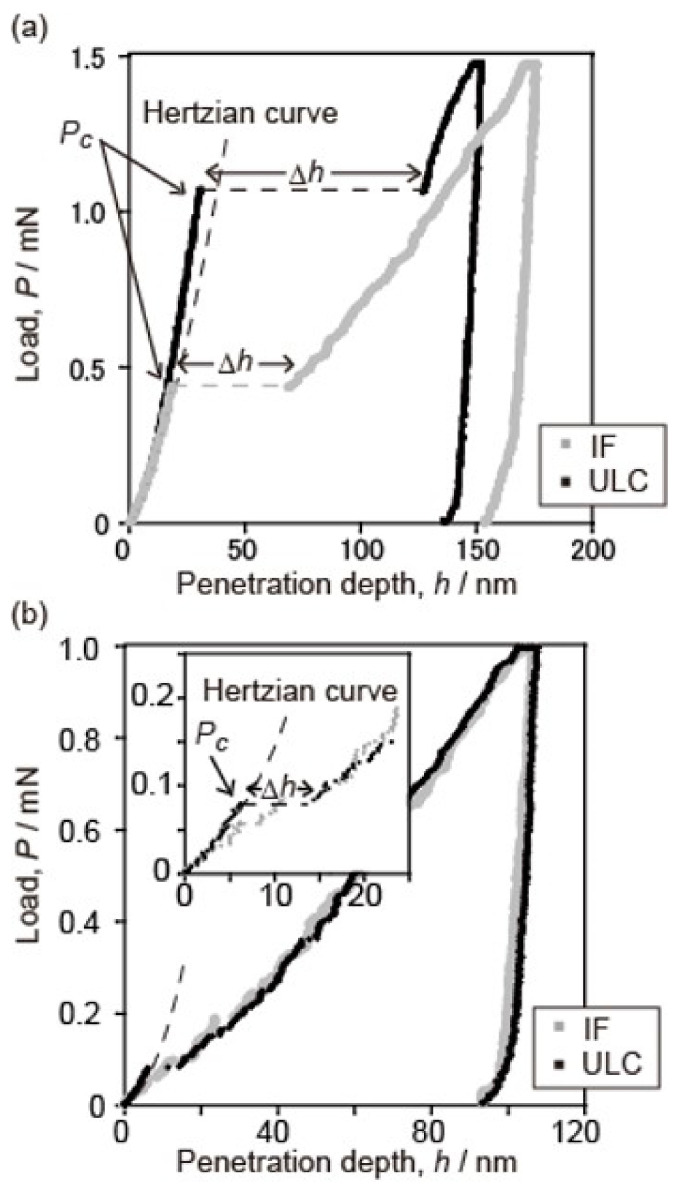
Typical load–displacement curves for (**a**) the low-density and (**b**) the high-density samples [120].

Sekido et al. also evaluated the effect of the pre-existing dislocation density in Fe–C steels [120]. Figure 9 shows scanning transmission electron microscopy (STEM) micrographs of interstitial free (IF) steels with different dislocation densities. The dislocation densities, *ρ,* of the tensile-strained and fully annealed samples were 10^14^ m^−2^ and 10^11^ m^−2^, respectively. Figure 10 shows typical load–displacement curves for low-density and high-density samples. A clear pop-in behavior appeared in the low-density sample shown in Figure 10a, while the pop-in behavior was not clear in the high-density sample, as shown in Figure 10b. The average spacing between the dislocations, which is given by 1/√*ρ*, were 0.1 μm and 3.2 μm for the high- and low-*ρ* samples, respectively. The typical penetration depth at which the pop-in started was approximately 30 nm, as shown in Figure 10, and the corresponding size of the high stress field was approximately 500 nm. Therefore, the stress field of the high-*ρ* sample probably included the pre-existing dislocations, leading to a very low *P*_c_, while the probability was relatively low in the case of the low-*ρ* sample, resulting in a high *P*_c_ value. Patel and Lee conducted spherical nanoindentations with different indenter curvatures on a sample with a certain dislocation density in W [124]. They showed that the pop-in critical stress decreased with an increasing tip radius, which can be understood by the single dislocation source model within a stress field. However, the pop-in stress with a larger tip radius was higher than that expected for the single dislocation source model. They discussed the reason for the collective operation of multiple dislocation sources, which may correspond to multipinned dislocation sources or local dislocation networks.

Pre-existing dislocations and their evolution during deformation is a crucial factor for mechanical behavior. We sometimes use a model of a constant dislocation density for a constant strain rate, but the density may vary in a practical condition. The evolution of the dislocation density depends strongly on an initial state and a generation potency. The generation potency consists of multiplication and nucleation of dislocation, therefore, the balance of them could be an important condition.

### 5.2. Solid Solution Element

The doping effect of solid solution elements on the pop-in behavior is an attractive issue as a point-defect-induced plasticity initiation. There are two main categories of doping elements; namely, H and other elements.

The H effect was first reported by Mine et al. [125]. They showed that the critical load for pop-in was decreased by the precharging of H to stainless steels, suggesting a lower critical stress for the activation of dislocations with in-solution H atoms. Barnoush et al. and Zamanzade et al. performed in situ electrochemical nanoindentation to maintain a constant H content in an austenitic stainless steel sample [126,127]. They also showed that H charging decreased the critical stress for pop-in and discussed the effect of a reduction in the elastic self-energy of dislocations for a homogeneous dislocation nucleation model. Tian et al. recently reported an interesting experimental study of the effect of H on the mechanical behavior of Zr-based bulk metallic glass (BMG) [128]. They showed that H charging increased the pop-in critical load, as well as Young’s modulus and the hardness of the BMG material, which is the antithesis of the trend in crystalline materials. They proposed a reason for the stabilization of the shear transformation zone (STZ) by H. The other topics of pop-in behavior in BMG materials are described in a later section.

**Figure 11:**
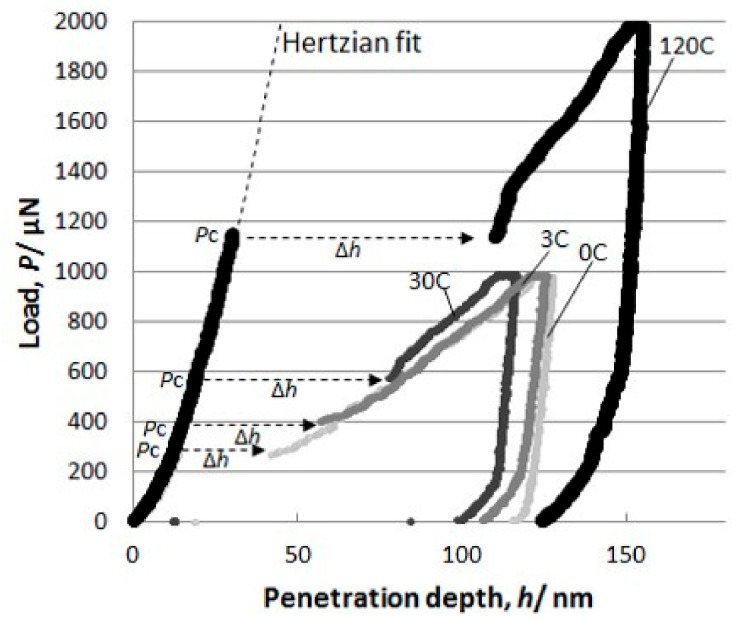
Typical load–displacement curves obtained by nanoindentation of Fe–C binary alloys with various C contents [132].

**Figure 12:**
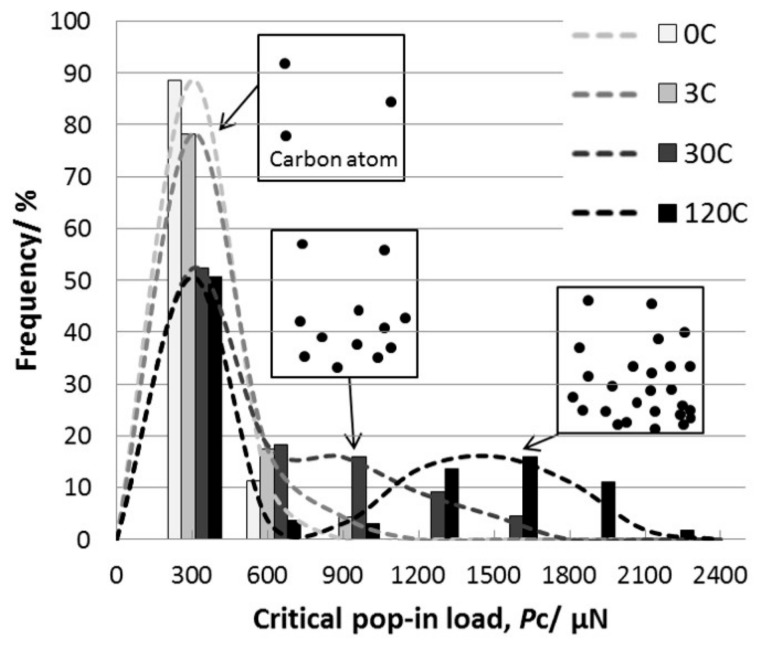
Probability distribution of *P*_c_ for each sample [132].

The effects of other alloying elements have also been reported in various materials. Bahr and Vasquez showed no alloying effect on the pop-in behavior of Cu–Ni alloys [129]. They also demonstrated the effect of the alloying element on the long-distance dislocation mobility, which affects the macroscopic hardness rather than the local dislocation nucleation. Le Bourhis and Patriarche investigated the doping effect of n- and p-type dopants for GaAs [130]. No dopant type remarkably affected the pop-in critical load, while the dislocation structure in the rosette arm was different with the different mobilities of the screw dislocations. This result suggests that the pop-in stress is not dependent on dislocation mobility. One of the key issues of the alloying effect is C in steel, because C content significantly affects the strength of the steel [131]. Figure 11 shows typical load–displacement curves obtained by nanoindentation for Fe–C binary alloys with C contents of 0, 3, 30, and 120 mass ppm (referred to as 0C, 3C, 30C, 120C) [132]. The critical load, *P*_c_, at which pop-in occurred increased with the concentration of the in-solution C. To clarify the variation in the deviation, the probability distribution of *P*_c_ for each sample is shown in Figure 12. The distribution of *P*_c_ is Gaussian-like at 0C and 3C, with a peak at approximately 350 mN. However, at a higher nominal C concentration, the peak height at approximately 350 mN decreased and another peak appeared at a higher load exceeding 500 mN. Additionally, the peak position shifted to a higher load and the peak width widened at 120C, compared to that of 30C. The pop-in phenomenon was controlled by the thermal activation process because both peaks are Gaussian-distributed regardless of the position of the peak. Accordingly, the thermal-activation process seems to be dominant for pop-in generation, even if the solid-solution C atom is related. As shown in Figure 12, the frequency distribution of *P*_c_ varied with the C concentration because as the C concentration increased, the peak at approximately 350 mN remained constant, while the other peak positions shifted to a higher load. This trend suggests a nonuniform C distribution. The peak at 350 mN corresponds to the behavior under C-free conditions because the peak appeared even in the 0C sample. If the spatial distribution of C atoms remained uniform after the in-solution C concentration increased, only the average value was expected to increase, while the distribution shape remained a single peak. Therefore, the multiple peaks suggest that different mechanisms dominate pop-in behavior. As the peak position at 350 mN was constant regardless of the C concentration and the peak was highest in the 0C sample, the mechanism was governed by the same resistance to dislocation nucleation, where in-solution C atoms were hardly involved. However, the peak at the higher load positions that appeared after the C addition was considered to be caused by the interaction between single or multiple in-solution C atoms and dislocations with higher resistance to dislocation nucleation.

The solution element effect for the dislocation nucleation is important especially when the pre-existing dislocations are not enough to assume a given strain. However, solid-solution strengthening in the conventional modeling is based on an interaction between pre-existing dislocations and in-solution elements, and thus may not be applicable to the nucleation mechanism. The alloying effect is a center of the metallurgy; therefore, the understanding of the fundamental effect is extremely important.

### 5.3. Grain Boundary

**Figure 13:**
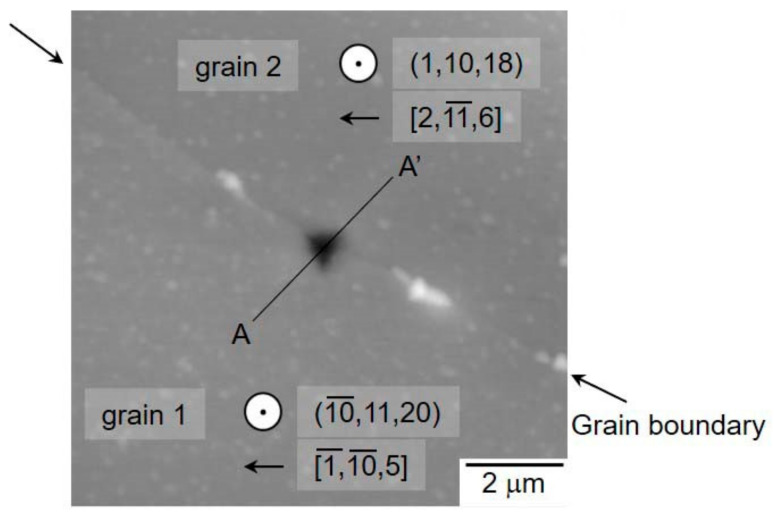
SPM image representing the triangle indent mark on the grain boundary with a high positioning accuracy [135].

**Figure 14:**
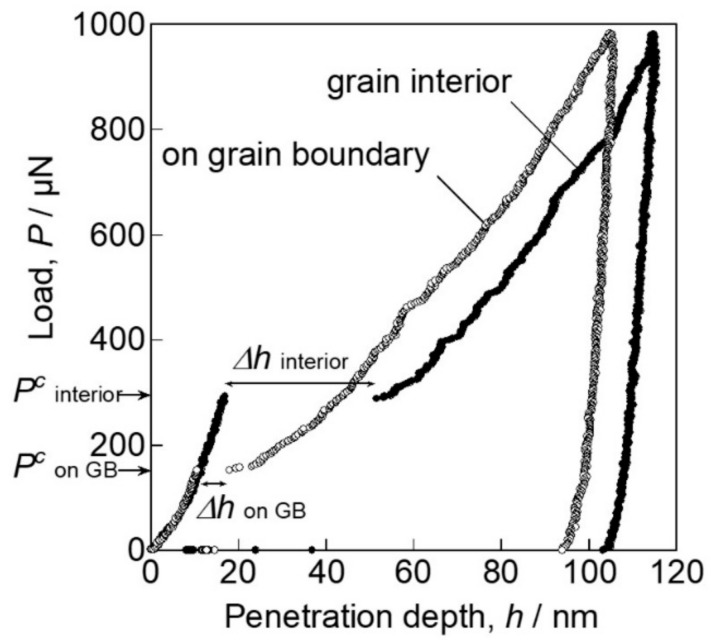
Typical load–displacement curves obtained by nanoindentation measurements just above the grain boundary and within the grain interior far from the grain boundary [135].

**Figure 15:**
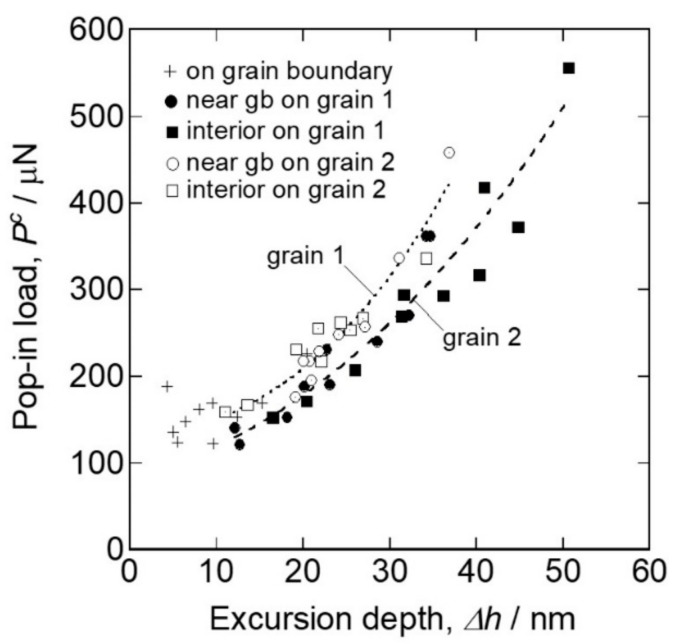
Relation between the critical load, *P_c_,* and pop-in depth, Δ*h* [135].

Grain boundaries significantly affect the strength of materials, and many researchers have studied the local mechanical behaviors by nanoindentation [133,134,135,136,137,138,139,140,141,142,143]. Chen et al. investigated the initiation behavior of nc-Cu [133]. They showed that the critical stress at the onset of plasticity was close to the theoretical strength, and suggested the activation of a dislocation source at the grain boundary. Yang and Vehoff performed a pop-in analysis for nc-Ni and showed that the critical load for the first pop-in did not depend on the grain size from 0.3 to 1.5 μm, indicating that the nucleation site was the grain interior [136]. The second or later pop-in was dependent on the grain size, suggesting the activation of a grain-boundary source by expanding the stress field. The first pop-in at the grain boundary was directly evaluated by probing an indenter exactly on a single grain boundary with a SPM for Ti-added IF steel [135,137]. Figure 13 shows the SPM image representing the triangle indent mark on the grain boundary with a high positioning accuracy. Figure 14 shows typical load–displacement curves obtained by the nanoindentation measurements just above the grain boundary and within the grain interior far from the grain boundary. Both cases showed a clear pop-in on the loading segment. Figure 15 shows the relation between the critical load, *P_c_,* and pop-in depth, Δ*h*. On the grain boundary, *P_c_* had relatively lower values (100–200 μN), whereas in the grain interior, it was dispersed up to approximately 600 μN. The results suggest that grain boundaries act as effective dislocation sources for enhancing the dislocation emission for plasticity initiation. Wang and Ngan showed another mechanism of pop-in associated with grain boundaries in Nb [134]. They demonstrated that the second large pop-in event occurred at a considerably higher load range, which corresponded to the activation of the grain-boundary source leading to slip transfer into the adjacent grain. Furthermore, the critical load for the grain boundary pop-in depended on the misorientation between the two grains. Khosravani et al. subsequently demonstrated the boundary pop-in in martensitic steel with a hierarchical microstructure [139]. They used several spherical indenters with different curvatures to clarify which boundary corresponds to the grain boundary pop-in in the hierarchical microstructure. They concluded that the grain boundary pop-in was attributed to the interaction of dislocations with lath boundaries and their transmission into the neighboring grain. Javaid et al. also recently showed the grain-boundary pop-in in W [143]. They also investigated the microstructures of dislocations and grain boundaries associated with indentation-induced deformation and demonstrated a remarkable grain-boundary movement, even at ambient temperature. Segregation of an alloying element on grain boundaries is an important structural factor in grain-boundary-associated behavior. Carbon in steels is a major issue, as described in the previous section. Several papers demonstrated that the segregation of C atoms at the grain boundary increased the critical stress of the grain-boundary pop-in [138,140,142]. These results are expected to provide important insights into the mechanisms of grain-boundary strengthening in macroscopic properties, such as the Hall–Petch model [144,145].

The dislocation source mechanism at grain boundaries should be revealed for an interpretation of the strengthening by a grain boundary because dislocation generation is the essential process of the slip transfer from one grain to the adjacent grain in polycrystalline materials. The critical stress for the activation and potency for generation of the source at grain boundaries may depend on the various factors including grain-boundary energy, atomistic structure, misorientation angle between the neighboring grains, crystallographic orientation of a grain-boundary plane, etc. Local mechanical characterization could be able to separate the factors and simplify the condition, leading to deeper knowledge of the grain-boundary strengthening.

## 6. Simulation

**Figure 16:**
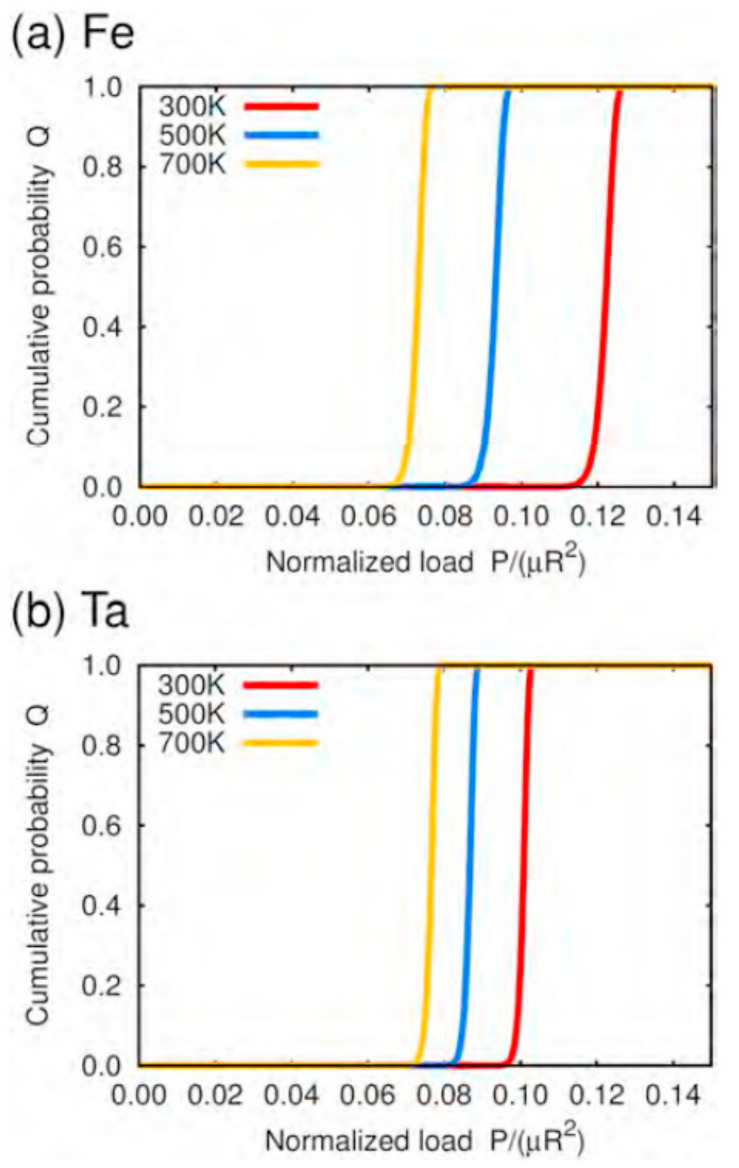
Predicted temperature dependence of the pop-in event in (**a**) Fe and (**b**) Ta [178]. The vertical axis is the pop-in cumulative probability, Q, while the horizontal axis is the normalized load (P, μ, and R are load, shear modulus, and indenter radius, respectively).

In addition to experiments and theory, computer simulations have become an important approach to unveil the origin of plasticity in various materials [146,147,148,149,150,151,152,153,154,155,156,157,158,159,160,161,162,163,164,165,166,167,168]. Several simulation techniques have been used, from atomistic to continuum to analyze plasticity in crystalline materials: density functional theory (DFT), MD, phase field, discrete dislocation dynamics, and the FEM. Atomistic simulations, such as DFT and MD, directly treat individual atoms, and thus they are powerful tools for revealing the origin of plasticity from the atomistic scale. The DFT method, based on quantum mechanics, accurately assesses the structural and chemical aspects of plasticity on an atomic scale. Ogata et al. performed DFT calculations for the affine shear deformation of a perfect crystal lattice to reveal the ideal shear strength in a variety of materials [169,170]. Nagasako et al. conducted DFT simulations to evaluate the ideal shear strength of bcc V, Nb, and Ta, and showed excellent agreement with the experimental estimation from nanoindentation in the case of Ta [171]. Classical MD simulations based on the interatomic model potentials can treat plastic deformation beyond the space-scale of DFT, and they have often conformed to the experimental results to support a thermally activated physical model. MD nanoindentations in metals have been conducted to realize atomic-scale observation of incipient plasticity and to discuss the criterion for dislocation nucleation [45,172,173,174,175,176,177]. Kelchner et al. performed MD simulations to show dislocation nucleation and discuss the defect structures detected by the centrosymmetry parameter [175]. Salehinia et al. calculated the effects of stacking faults, crystallographic orientations, and indenter sizes on the stochastic behavior of dislocation nucleation [45]. Meanwhile, Li et al. and Zhu conducted MD and FEM simulations to model incipient plasticity at an ideal stress level, and applied an instability criterion to the defect nucleation event [176,177]. These studies focused on the crystallographic and mechanical aspects of the dislocation nucleation events. In recent years, Sato et al. performed MD simulations and nudged elastic band (NEB) simulations for bcc Fe and Ta [178]. NEB is a type of static approach for the activation event that finds a minimum energy path and the saddle point of the path. They showed indentation-induced nucleation of shear dislocation loops with the minimum energy path of a saddle-point value close to 78.1 eV for Fe and 78.2 for Ta, which coincided well with the previous atomic-scale nanoindentation experiment for Au [107]. The estimation of the activation barrier enables us to discuss the time and temperature dependence (that is, dynamical aspect) of a physical event in plasticity beyond the general MD timescale based on the transition-state theory [179]. Sato et al. predicted the temperature dependence of the pop-in cumulative probability based on the atomically obtained energy barrier for dislocation nucleation, as shown in Figure 16 [178]. MD simulations have been utilized to evaluate the effects of lattice defects, such as vacancies [77,180], surface step [181], and misfit dislocations in the *γ/γ′* phase interface and void [182], on the incipient plasticity in nanoindentation frameworks. Discrete dislocation dynamics [183,184,185], phase fields [186], finite element simulations [187,188], and multiscale simulation approaches [189,190,191] have also been applied to reveal plastic deformation in nanoindentation far beyond the atomic scale.

The simulations have provided various information on plasticity for decades. As mentioned above, the pop-in event in crystalline materials is correlated with the generation of lattice defects, and it is also affected by pre-existing lattice defects. Therefore, accurate computation of lattice defects is significant to treat the pop-in event in a simulation framework. Further development of the simulation technique and computer performance should widen the target of the simulation and provide fundamental knowledge of the pop-in and related phenomena.

## 7. Other Mechanisms of the Event

The indentation-induced serrated flow in BMG is an attractive behavior in the deformation mechanisms of macroscopic properties [192,193,194,195,196,197,198,199,200,201,202,203,204,205,206,207,208]. The homogeneous and inhomogeneous deformation behaviors in metallic glasses are described in the pioneering works by Spaepen [192] and Argon [193]. The macroscopic behaviors are discussed based on the diffusive and displacive models in an atomic scale. The recent nanomechanical characterization can approach to the issues experimentally. Schuh and Nieh [194] performed nanoindentation measurements on BMGs to analyze the fundamental deformation mechanism. They demonstrated that the pop-in phenomenon in BMG corresponded to the formation of a shear band, and the event probability depended on the kinetics of the shear-band formation. That is, the pop-in occurred with a single shear-band formation when the strain rate was low enough, whereas no pop-in with multiple formations with continuous events appeared when the strain rate was higher because the given strain cannot be assumed by a single shear band. Mukhopadhyay et al. also demonstrated a pop-in event associated with shear-band formation, which was confirmed by AFM [195]. They showed a discontinuous strain rate and the peak of the rate corresponded to shear-band formation, suggesting an intermittent plastic flow in the BMG. Wang et al. measured three BMGs with different indenter shapes [197]. They showed multiple pop-in events on the loading curve and the resistance to plastic shear deformation was not sensitive to the indenter shape, but depended on the shear modulus of the materials. Kim et al. demonstrated the effect of alloying elements on the mechanical behavior of Fe-based BMGs [199]. The pop-in event probability depended on the alloy content and the mechanisms of shear-band formation associated with the free volume in the BMG. Limbach et al. investigated the effect of Al alloying on CuZr BMG [202]. Al alloying reduced shear localization as a transition from inhomogeneous to homogeneous plastic flows. They analyzed the thermally activated model based on the first pop-in probability, and concluded that the barrier energy for the initiation of the STZ increased with Al content, leading to a lower number and reduced size of the pop-ins on the load–displacement curves. This review paper does not include polymers, even though many applications of nanoindentation have been made. Some representative papers are referred for further discussion of the deformation mechanisms in the polymeric glasses [206,207,208].

Indentation-induced phase transformation is an interesting behavior for various materials. The structural phase transition in Si and Ge is a major topic [209,210,211,212,213,214,215,216]. Bradby et al. conducted indentation to demonstrate the behavior of the pop-in on a loading segment, as well as pop-out on an unloading curve with a drastic recovery in depth, and observed the cross-sectional microstructures beneath the indenter in Si [209,210]. They claimed that the pop-in on the loading curve corresponded to the transition into the other phase, and the pop-out on the unloading curve was associated with the transition into less dense phases. Additionally, the phase after the pop-out depended on the unloading rate, suggesting kinetic domination. Oliver et al. demonstrated a similar phase transformation behavior in Ge [216], while Bradby et al. showed no phase transformation in GaAs, InP, and GaN, but a slip deformation for the pop-in behavior [217]. Shape-memory alloys also exhibit transformation-associated pop-in behavior [218,219,220,221]. Caër et al. demonstrated the pop-in and pop-out behavior in CuAlBe and discussed the phase transformation and deformation mechanisms [218]. Laplanche et al. suggested the occurrence of twin formation and phase transformation in NiTi alloys [220]. The phase-transformation-induced plasticity (TRIP) effect is expected to be a novel idea for obtaining a better performance in strength–ductility balance in steel, and many studies have approached this behavior by indentation [222,223,224,225,226,227,228,229]. Lu et al. analyzed the indentation-induced mechanical behavior of retained austenite in a bearing steel [222]. They showed that the pop-in phenomenon corresponded to the strain-induced martensitic transformation, and the stability of the austenite phase depended on the local C content. Fe–C alloys and Fe–Ni alloys were analyzed to clarify the stabilizing factors of the austenite phase, including a constraint by the surrounding harder phases of the as-quenched and/or tempered martensite phases [226,227].

**Figure 17:**
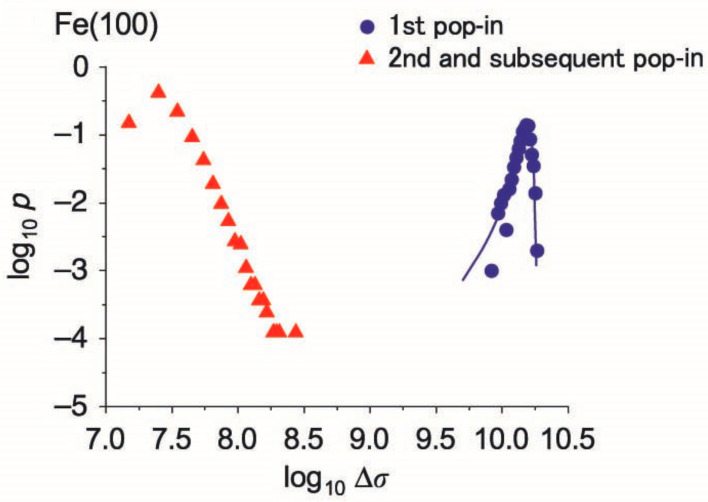
Probability distribution of pop-in magnitude in bcc Fe [235].

After the first pop-in event with indentation-induced deformation behavior, the second or later pop-in occurs significantly in some materials, especially fcc metals, and the behavior is characterized as a repetitive plastic deformation with an interval of elastic deformation [25]. The repetitive event is observed as serrations in uniaxial stress testing, and the phenomenon is modeled with respect to the dislocation avalanche [230,231,232,233,234,235]. Figure 17 shows the probability distribution of the pop-in magnitude in bcc Fe [235]. For the first pop-in event, the distribution shows a Gaussian-like shape, as discussed in previous papers on thermally activated processes. However, the second and subsequent events follow the power-law function with a fractal feature, which is a completely different physical model. These results suggest that the intermittent plasticity after the first pop-in includes the complicated mechanism of local and macroscopic deformation of materials.

The distinct strain burst as pop-in is associated with not only dislocation nucleation at defect-free volume in crystalline materials, but also the local diffusive behavior in BMGs. Stress/strain-induced phase transformation is often reflected on the load–displacement curve, which gives us a chance to reveal the mechanisms of stress-assisted thermomechanical behavior. The intermittent plasticity attracts the various fields of people including solid-state physics, and the instability phenomenon may lead a new model of the plasticity.

## 8. Summary and Future Perspective

This paper reviewed the attractive pop-in phenomenon in a number of studies with experimental, computational, and theoretical approaches. Attention to this phenomenon is still growing from both the engineering and scientific views. In the engineering sense, the three remaining issues are presented herein. First, the pop-in phenomenon could be an elementary step in yielding behavior. A conceptional consensus of “microyielding” behavior prior to macroyielding exists, but doubts of the initiation site, critical local stress for the initiation, and subsequent evolutional mechanisms for macroscopic yielding still remain. Second, pop-in occurs at an extremely high stress close to the theoretical strength, which is presumably analogous to the condition in the vicinity of the crack tip with a stress intensity and plaston concept under a mechanically excited state [236]. Local plasticity under high stress is a significant issue, particularly in crack initiation and propagation. Third, pop-in behavior might reveal the strengthening mechanisms by grain boundaries because slip transfer at a grain boundary may include a step of dislocation nucleation/generation at a source in the vicinity of the grain boundary. Grain-boundary strengthening is a significant issue from an engineering perspective because the strengthening factor can improve both strength and ductility simultaneously, which are generally in a trade-off relationship. The other three issues are also described physically. First, the mechanism of dislocation nucleation at a small volume is still unclear, especially in terms of homogeneous or heterogeneous nucleation. Even though heterogeneous nucleation dominates the behavior, the question remains unsolved for the heterogeneity in the crystal. Second, the evolution of the high-density dislocation structure upon pop-in is unclear. Because the dislocation structure includes a very complicated state with the activation of multislip systems, the evolution mechanism could include a multiplication source formed by dislocation–dislocation interaction and/or other mechanisms. Third, pop-in can be recognized as an unstable plasticity phenomenon, similar to a dislocation avalanche. Because the intermittent plasticity is composed of consecutive events of the phenomenon, the general model of a mechanical equilibrium may not be applicable to plastic deformation in some cases. The mechanical characterization on a very small scale has great potential to address these issues, and another subsequent achievement is required in the near future.

## Data Availability

No new data were created or analyzed in this study. Data sharing is not applicable to this article.
